# Tackling Antimicrobial Resistance with Small Molecules Targeting LsrK: Challenges and Opportunities

**DOI:** 10.1021/acs.jmedchem.0c01282

**Published:** 2020-11-05

**Authors:** Pasquale Linciano, Valeria Cavalloro, Emanuela Martino, Johannes Kirchmair, Roberta Listro, Daniela Rossi, Simona Collina

**Affiliations:** † Department of Drug Sciences, University of Pavia, Viale Taramelli 12, 27100 Pavia, Italy; ‡ Department of Earth and Environmental Science, University of Pavia, Via Sant’Epifanio 14, 27100 Pavia, Italy; § Department of Pharmaceutical Chemistry, Faculty of Life Sciences, University of Vienna, 1090 Vienna, Austria

## Abstract

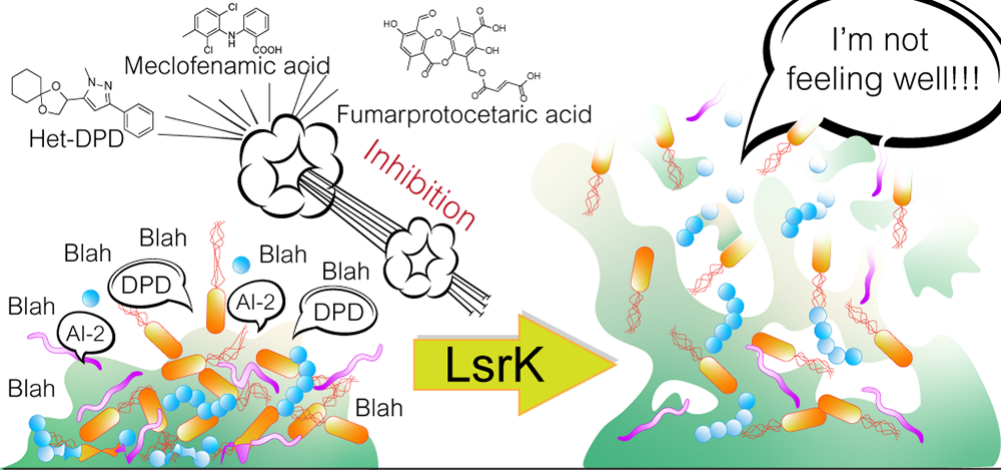

Antimicrobial resistance (AMR) is a growing threat with severe health and economic consequences. The available antibiotics are losing efficacy, and the hunt for alternative strategies is a priority. Quorum sensing (QS) controls biofilm and virulence factors production. Thus, the quenching of QS to prevent pathogenicity and to increase bacterial susceptibility to antibiotics is an appealing therapeutic strategy. The phosphorylation of autoinducer-2 (a mediator in QS) by LsrK is a crucial step in triggering the QS cascade. Thus, LsrK represents a valuable target in fighting AMR. Few LsrK inhibitors have been reported so far, allowing ample room for further exploration. This perspective aims to provide a comprehensive analysis of the current knowledge about the structural and biological properties of LsrK and the state-of-the-art technology for LsrK inhibitor design. We elaborate on the challenges in developing novel LsrK inhibitors and point out promising avenues for further research.

## Introduction

Antimicrobial resistance (AMR) and the worldwide increase of superbug infections are recognized by the World Health Organization (WHO) as global concerns for public health and healthcare systems’ sustainability.^1,2^ AMR infections cause approximately 700 000 deaths annually, and they are expected to become the leading cause of death by the year 2050, especially in low- and middle-income countries.^3−5^ Similarly, it is projected that in the year 2050, AMR could lower the global gross domestic product by up to one trillion dollars annually.^6^ To challenge this inauspicious outcome, in 2015, the WHO launched the Global Antimicrobial Resistance Surveillance System (GLASS).^7^ The primary aim of GLASS is to foster global, national, and regional actions to support AMR’s spread surveillance and research.^2,8^ Thus, five strategic objectives were set out: (i) promotion of initiatives for raising awareness about this issue, (ii) optimization of the use of antibiotics in both human and animal health, (iii) delineation of global strategies to monitor and contain the spread of resistance, (iv) application of preventive measures to reduce the incidence of infections, and (v) incentivization of investments in the research of new pharmaceutical tools and medicines.^7^

Overuse, inappropriate prescription, and extensive agricultural use of antibiotics have exposed bacteria to intense, selective evolutive pressure. This led to the development of protective mechanisms to inactivate, remove, and, in general, circumvent the toxicity of the antibiotics against bacteria.^8−11^ These mechanisms of resistance exploit the reduction of drug permeability,^12^ the excretion of the antibiotic through active efflux pumps,^13^ the production of antibiotic-inactivating enzymes (i.e., β-lactamases),^14−16^ or the formation of biofilms,^17^ thus conferring reduced susceptibility to antibiotic activity.

Biofilm formation is the typical virulence mechanism by which bacteria organize in communities, and it is characterized by (i) an extracellular matrix that envelops the microorganism, (ii) the presence of different types of organisms (eukaryotic and prokaryotic), and (iii) an anchoring surface of aggregate colonial bacteria.^17,18^ With resistance at the cellular level, biofilms confer additional resistance to bacteria, commonly referred to as community resistance. It is estimated that about 80% of all human bacterial infections are complicated by the formation of biofilms, where bacteria can have a 1000-fold higher tolerance to antibiotics than the same organisms in a planktonic state.^19,20^

The assemblage and “social” organization in a biofilm require bacteria to communicate with the neighbors in order to coordinate efforts and accomplish cooperative activities. The quorum sensing (QS) signaling is the most effective known cell-to-cell mechanism that bacteria, both Gram-positive^21,22^ and Gram-negative,^23^ used to communicate, coordinate and act as a population, thereby gaining some benefits that otherwise were unattainable.^24^ Moreover, recent studies indicate that QS can also conversely coordinate the dispersion of the biofilm. This process is activated when nutrients and resources within the biofilm become limited or waste/toxic products accumulate, allowing bacteria to escape from the colony and populate new areas.^25,26^

Besides biofilm formation, QS processes are diverse and depend on the communities’ specific needs. Thus, QS may trigger changes in bacteria’s physiology,^23,27,28^ inducing modification in antibiotic susceptibility,^29^ virulence factor production,^30,31^ symbiosis, competence,^32^ bioluminescence,^33,34^ motility, cellular division control,^35^ sporulation,^36^ and genetic transfer (transformation, conjugation, and transduction).^37^ Quenching the QS response thus represents an attractive therapeutic strategy for the treatment of AMR infections. The first experimental evidence of quorum quenching’s potential in counteracting the bacterial resistance mechanism dates back to the 1990s. Compound 4-bromo-3-butyl-5-(dibromomethylene)furan-2(5*H*)-one was discovered to be able to interfere with the *N*-acyl homoserine lactones (AHL)-mediated QS both in vitro and in vivo,^38^ paving the way to the identification and development of several small molecules with anti-QS activity.^39−44^ Analogously, mutations on genes involved in QS significantly reduced mutant bacteria’s virulence and their capability to form a biofilm.^45,46^

Through modulation/inhibition of QS, several bacterial virulence factors that facilitate human infections can be controlled, and their harmful effects, including mortality, can be reduced. Importantly, because these virulence factors are not essential to bacterial growth and survival, a treatment that does not inhibit bacterial growth will not generate selective pressure. Therefore, the risk of resistance formation can be reduced substantially.^23,47−51^ Thus, interfering with the QS has become an appealing strategy to prevent the spread of AMR.^52,53^

## The Key Role of LsrK Kinase in the AI-2 Mediated QS Cascade

QS enables bacteria to recognize population density by measuring the accumulation of nonspecific signaling molecules secreted by community members. Only when the population density is high will the accumulation of the signal in the extracellular environment be suitable to activate the response. The messengers of QS are the so-called autoinducers (AIs).^23,54^ Conventionally, AIs have been divided into three main categories: (i) *N*-acyl homoserine lactones (AHLs),^54−56^ exploited by Gram-negative bacteria, (ii) oligopeptides,^57^ exploited by Gram-positive bacteria,^58^ and (iii) autoinducer-2 (AI-2), exploited by both Gram-positive and Gram-negative bacteria.^59^ Other QS signals include the (iv) *Pseudomonas* quinolone signal (PQS),^60,61^ (v) diffusible signal factor (DSF),^62,63^ (vi) γ-butyrolactone,^64^ (vii) 2-amino acetophenone (2-AA),^65^ and (viii) bradyoxetin.^66^

AI-2 signaling differs from all other QS strategies because it allows for interspecies communication and has been defined as “universal language.”^59^ The first evidence of the AI-2-mediated signal date back to 1994, when QS activity was observed in bacterial strains lacking the AHL synthase.^34^ A few years later, AI-2 activity was detected in a wide range of LuxS-containing species, confirming the role of AI-2 as QS signaling molecules.^67^ At present, the synthase responsible for the biosynthesis has been detected in more than 70 bacterial species.^68,69^

**Figure 1:**
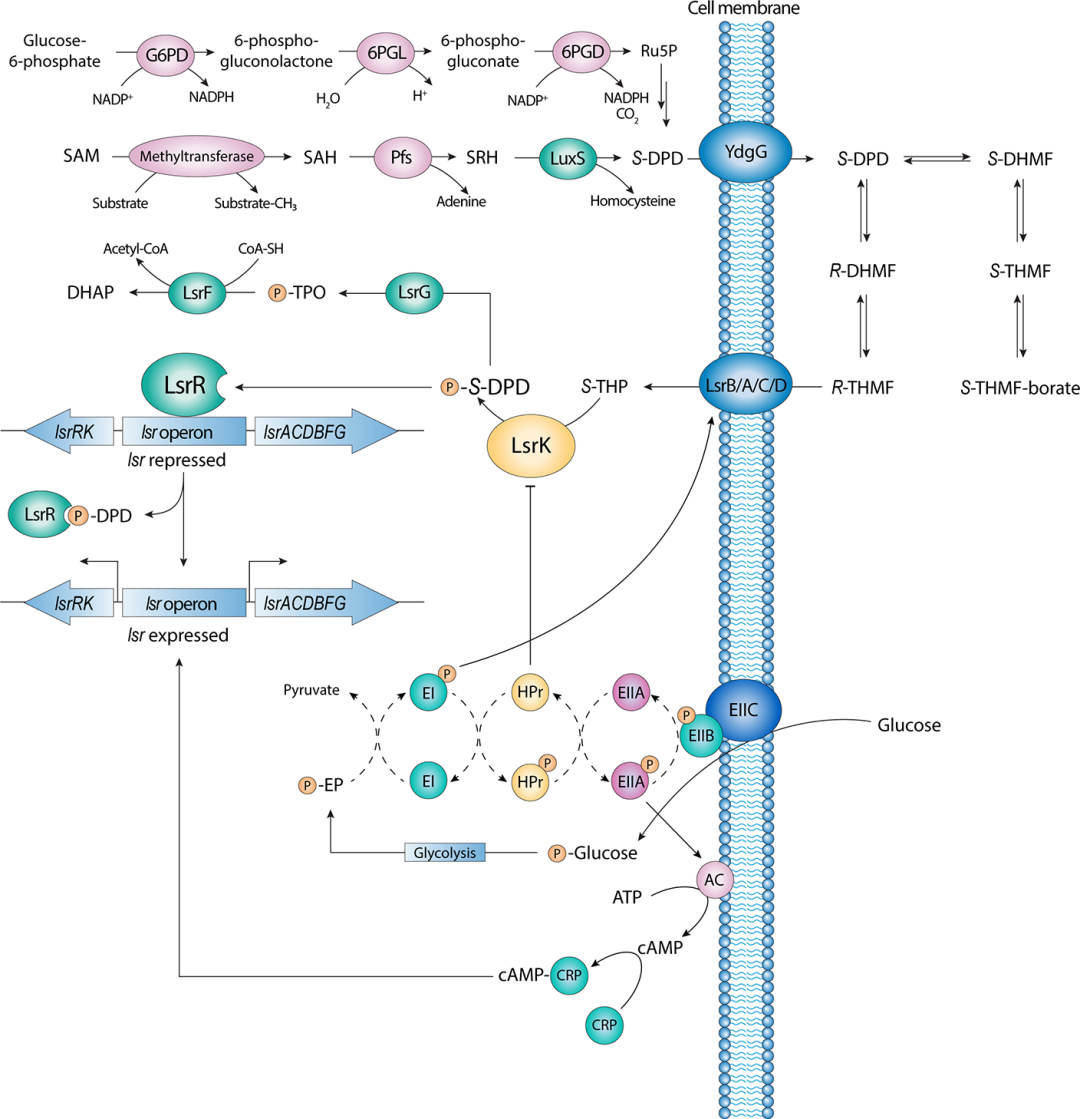
AI-2 mediated QS cascade and its entanglement with the carbon catabolite repression (CCR) and the phosphoenolpyruvate (PEP)-dependent sugar phosphotransferase system (PTS).

**Figure 2:**
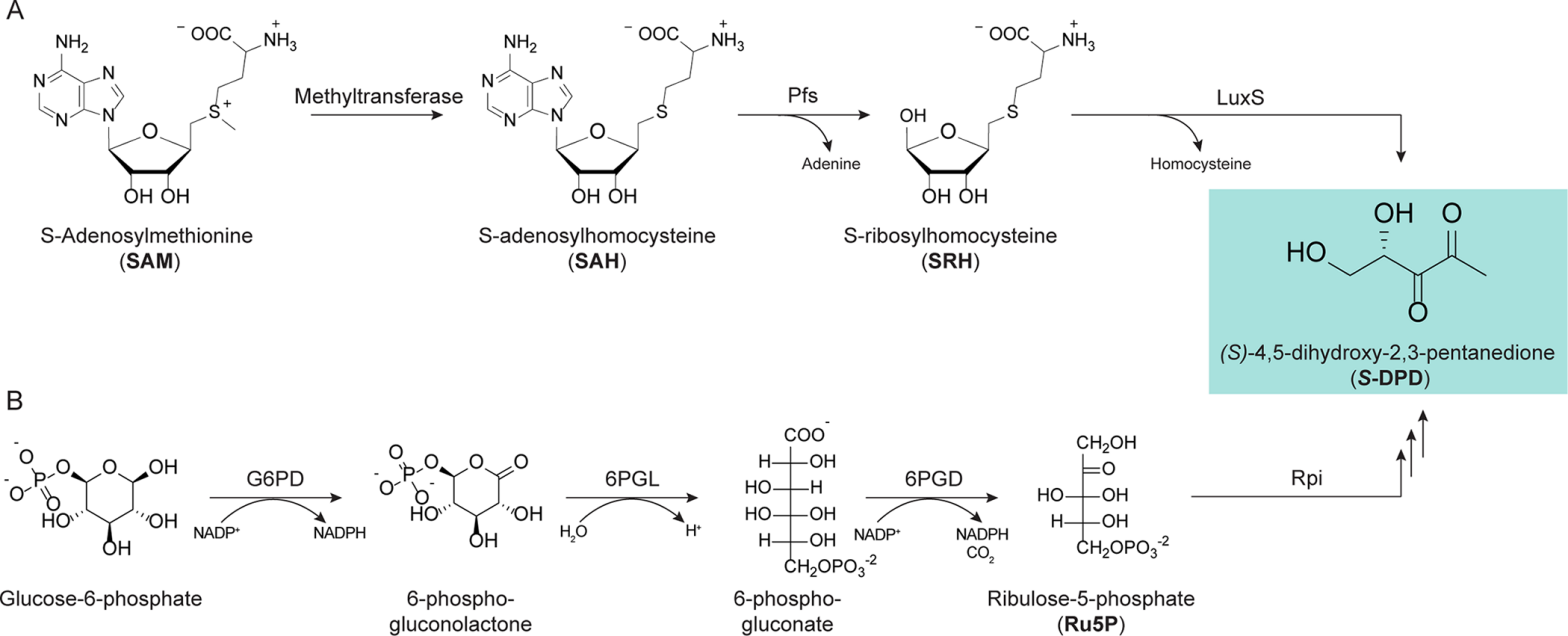
Biosynthesis of AI-2 via OPP pathway.

All AI-2 compounds share the 4,5-dihydroxy-2,3 pentanedione (DPD) as a common precursor. DPD is biosynthesized in a three-step pathway (Figure 1). In the first step, *S*-adenosylmethionine (SAM) is demethylated by a methyltransferase to generate *S*-adenosylhomocysteine (SAH). Because SAH is a potent inhibitor of the methyltransferases itself, in the second step, it is quickly degraded by Pfs (a 5′-methylthioadenosine nucleosidase, MTAN) through the removal of the adenine moiety to form *S*-ribosylhomocysteine (SRH). In the third step, an *S*-ribosylhomocysteinase (LuxS) catalyzes the displacement of the homocysteine moiety from SRH to release AI-2 (Figure 1 and Figure 2A).^70−73^

An alternative pathway for the biosynthesis of DPD requires the isomerization (by ribulosephosphateisomerase, Rpi) of d-ribulose-5-phosphate (Ru5P), which results from the catabolism of glucose via the oxidative pentose phosphate (OPP) pathway (Figure 1 and Figure 2B).

**Figure 3:**
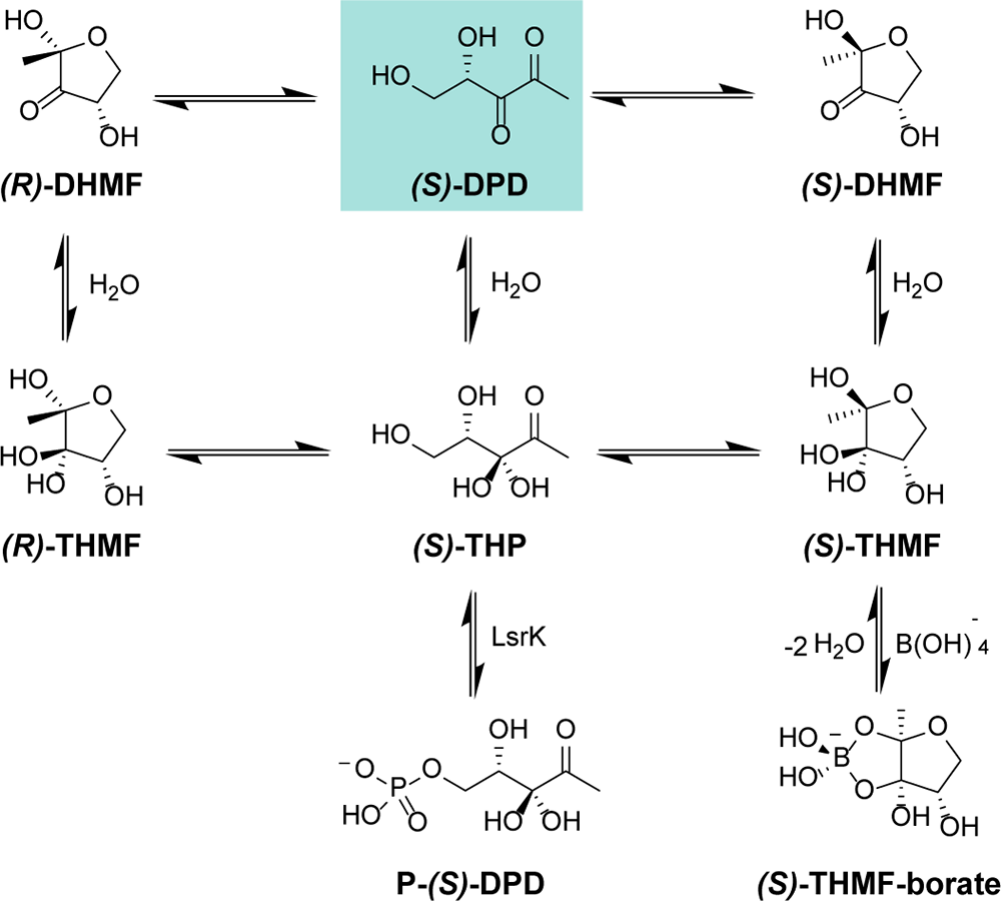
Equilibrium species of AI-2 in an aqueous environment.

However, DPD is not an effective AI-2. DPD itself has never been observed in vitro by MS or NMR analysis. Although DPD is more stable than other autoinducers (such as AHLs and oligopeptides), structural analysis in an aqueous solution of DPD analogues confirmed a complex equilibrium of structurally related compounds. DPD is a highly reactive molecule against electrophiles, and in an aqueous solution, it may undergo a spontaneous cyclization reaction, rearranging in a complex equilibrium of 4-hydroxy-5-methyl-3(2*H*)-furanone (HMF) derivatives. Linear DPD is in equilibrium with its two cyclic isomers, *S*-DHMF and *R*-DHMF (Figure 3). Their hydration at C3 results in the two cyclic, tetrahydrate isomers, *S*-THMF and *R*-THMF (Figure 3). This hypothesis was confirmed when the first AI-2 was cocrystallized in complex with *Vibrio harveyi* LuxP, revealing the chemical structure of the *S*-isomer of THMF in the form of borate diester (*S*-THMF-borate).^74,75^ In support of this evidence, (*R*)-THMF was cocrystallized with the LsrB transporter protein of *Salmonella typhimurium*, *Sinorhizobium meliloti*, and *Yersinia pestis*.^76−78^ These results led to the currently accepted model that AI-2 is a set of equilibrium forms of DPD rather than a single molecule. Different forms of AI-2 are manifested under varying environmental conditions.

Although there is evidence supporting the nonenzymatic, spontaneous conversion of DPD to various equilibrium forms of AI-2, a recent study has indicated the presence of alternative synthetic pathways.^79^

Bacteria can selectively recognize the diverse DPD-derivatives triggering specific QS cascades. Moreover, DPDs can be detected by different bacterial species, justifying the adaptable nature of these molecules as universal messengers among bacteria.^74,80^

Once biosynthesized, AI-2 is actively released in the extracellular space by the proposed YdgG protein, although other unknown mechanisms could be present (Figure 1).^81^ Once a threshold concentration of AI-2 in the extracellular environment is reached, *R*-THMF is internalized via the Lsr (LuxS regulated) transporter system, an ATP-binding cassette.^77,82^ In the cytoplasm, *R*-THMF, in equilibrium with the hydrated linear DPD (*i.e.*, *S*-THP, Figures 1–2), is further phosphorylated at position 5 by LsrK. Phosphorylation by LsrK drives the equilibrium to the tautomeric form in the linear conformation, producing *S*-THP-phosphate (commonly also known as phospho-DPD or P-DPD, Figure 1).^77,83^ The phosphorylation of DPD is the crucial step in the triggering of the QS cascade. P-DPD binds to the transcriptional repressor LsrR, which dissociates from the promoter region of the two divergently transcribed *lsrACDBFG* and *lsrRK* operons. As a result, *lsrACDBFG* transduces the transporters LsrA/C/D, which leads to an increase of the internalization of the signal molecules and, consequently, in sustaining the QS cascade (Figure 1). The activity of LsrR as a repressor of both operons is under negative autoregulation feedback control. When the level of P-DPD within the cell is low, LsrR, in its active state, represses the expression of both the *lsr* and *lsrRK* operons, thus reducing the production of LsrR itself.

Conversely, in the presence of AI-2s, LsrR is inhibited. The transcription of the two operons is activated, leading to speed up the production of all enzymes and transporters involved in the AI-2 mediated QS cascade. This mechanism of autoregulation of LsrR allows the cells of bacteria to respond quickly to the extracellular level of AI-2. Accordingly, with the described mechanism of action, mutants that do not express LsrK cannot activate *lsr* transcription, resulting in reduced expression of the Lsr transporter and extracellular AI-2 accumulation.^84^ At the end of its life cycle, P-DPD is degraded by LsrG and LsrF. LsrG catalyzes the isomerization of P-DPD into 3,4,4-trihydroxy-2-pentanone-5-phosphate (P-TPO). LsrF is a thiolase that catalyzes the transfer of an acetyl group from hydrated P-TPO to coenzyme A, releasing dihydroxyacetone phosphate (DHAP) and acetyl-CoA (Figure 1).^85^ Accordingly, *lsr* expression is increased in LsrG and LsrF mutants as a result of phospho-AI-2 accumulation.^85,86^

Several studies have been performed to better understand LsrK activity and its importance in bacterial QS. LsrK mutants do not activate *lsr* transcription because of the lack of phospho-AI-2, and, as a consequence, the reduced expression of the Lsr transporter results in extracellular AI-2 accumulation.^87^ Furthermore, when LsrK and ATP were added *ex vivo* (*i.e.*, in the extracellular medium) to *Escherichia coli*, *Salmonella typhimurium*, or *Vibrio harveyi* cultures (both in pure cultures and in a synthetic ecosystem), the phosphorylation of AI-2 outside the cells impedes the transport of phospho-AI-2 through the Lsr transporter due to its negative charge. As a result, a reduction in *lsr* expression and QS attenuation was observed (both in the single cultures and in these three species).^88^ Exploiting this observation, Rhoads et al. recently synthesized a functionalized biopolymer capsule of alginate and chitosan containing ATP. LsrK, modified with a C-terminal tyrosine tag, was covalently attached to the surface of the capsule. The addition of these capsules in the supernatants of *E. coli* cultures led to a modulation of the QS activity. Thus, these functionalized biopolymers with LsrK, and in a broad sense, other bacterial kinases, may represent a suitable strategy to adopt in human wound dressing to prevent wound infections by the quenching of AI-2 mediated QS activity.^68^ Taken together, these findings suggest that LsrK is an attractive anti-infective target, and they underline how the selective modulation of LsrK could attenuate AI-2 related pathogenesis.

Besides, recent studies have demonstrated the interconnection and control of AI-2 mediated QS with the availability of carbohydrates (e.g., glucose) and their catabolism processes.^89^ The transcriptional activity of *lsr* operons is directly controlled by carbon catabolite repression (CCR) by the cyclic AMP (cAMP)-CRP complex. (cAMP)-CRP modulates the transcription of *lsrRK* and *lsr* operon by binding to specific promoter sequences (Figure 1). (cAMP)-CRP works in tandem with the LsrR repressor to regulate AI-2 uptake. Conversely, the AI-2 mediated QS can be indirectly modulated by the phosphoenol-pyruvate (PEP)-dependent sugar phosphotransferase system (PTS) (Figure 1). PTS comprises three units: EI, HPr, and EII. The phosphorylation of EI seems to be necessary for the initial uptake of AI-2, although the exact mechanism remains to be determined. Interestingly, crystallographic studies performed by Ha et al. revealed that HPr could directly regulate the activity of LsrK by binding with the kinase.^89^ LsrK activity is inhibited when bound to HPr, indicating new linkages between QS activity and sugar metabolism. Therefore, a strong relationship between substrate availability, cell metabolism, and QS processes have been proved, together with the essential role of LsrK is in the QS process.^87,90^

## Structure and Catalytic Activity of LsrK

**Figure 4:**
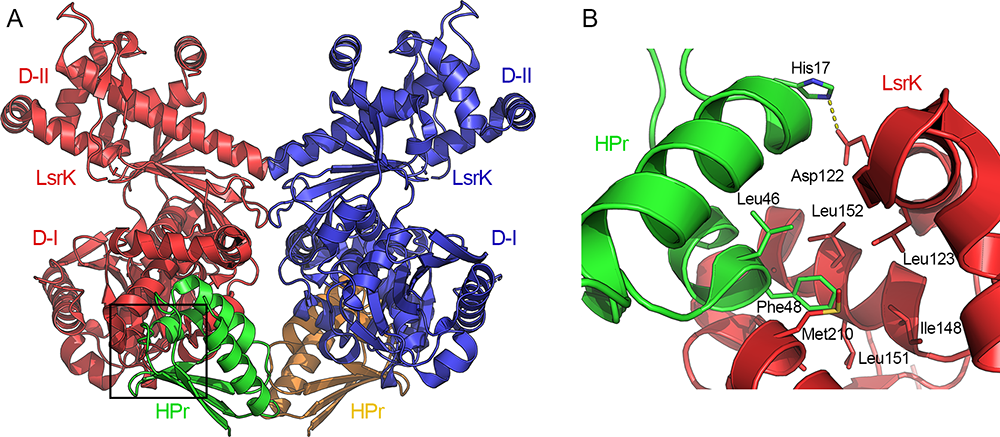
(A) Crystal structure of the LsrK-HPr-ATP complex (PDB 5YA1). The two subunits of LsrK are visualized as red and blue cartoons; the two subunits of HPr are displayed as green and ocher cartoons. (B) Close-up on the residues of HPr (green cartoon) involved in the interaction with LsrK (red cartoon). Amino acid residues are reported in stick mode. Yellow dashed lines represent the hydrogen and coordination bonds. Heteroatoms are color-coded (oxygen atoms in red, nitrogen atoms in blue, sulfur atoms in yellow).

From a structural point of view, LsrK belongs to the FGGY carbohydrate kinase family and catalyzes a phosphate group’s transfer from ATP to AI-2. Members of this family are widely found in bacterial genomes, and they are involved in the catabolic pathway of carbohydrates. Although discovered in the year 2003 already, LsrK was cloned, for the first time, in 2017.^91^ The first three-dimensional structures became available in the following year when Ha et al. reported the first crystallographic structures of *Escherichia coli* LsrK (*Ec*LsrK) cocrystallized with the HPr protein of the phosphotransferase system (PTS).^89^ The structures published by Ha et al. include a binary complex of LsrK-HPr at a resolution of 3.00 Å (PDB 5YA0), a ternary complex of LsrK-HPr-ADP at a resolution of 2.70 Å (PDB 5YA2), and a ternary complex of LsrK-HPr-ATP at a resolution of 2.70 Å (PDB 5YA1). The three reported crystallographic structures are similar in terms of structure. Each crystallographic unit consists of two nonsymmetrically arranged molecules of the LsrK-HPr complex (Figure 4A). The two subunits form ionic interactions mediated by two phosphate ions and the side chains of the two specular residues Lys204. However, exclusion chromatography coupled with light scattering analysis suggests that the functional unit of the LsrK-HPr complex may consist of a single monomeric complex.^89^

As observed in the X-ray structure of the three LsrK-HPr complexes, the protein–protein interaction between the two binding partners is dominated by hydrophobic interactions between Phe48 and Leu47 (to a lesser extent) of HPr, and the hydrophobic pocket of LsrK formed by Leu123, Ile148, Leu151, Leu152, Ala155, Tyr162, Met210, and Ala211 (Figure 4B).^89^ Phe48 and Leu47 are highly conserved in Gram-negative bacteria, and corresponding hydrophobic residues have been identified in Gram-positive bacteria (e.g., Ile47 and Met48). Besides, the ionic interaction between His15 of HPr and Glu122 of LsrK might represent the main “switch” for the control of the LsrK activity by HPr. His15 is the residue that mediates the phosphate group’s transfer from phosphorylated HPr (P-HPr) to the PTS’s EIIA protein. In its phosphorylated form, P-HPr carries the phosphate group bound to N^δ1^ of His15.^92^ Thus, Ha et al. proposed that when HPr is in its phosphorylated state, the steric clash and unfavorable repulsive ionic interactions induced by the phosphate group of P-HPr His15 and the negative charge of Asp122 of LsrK might prevent the formation of the LsrK/HPr complex, representing the junction point between the QS activity and sugar metabolism.

**Figure 5:**
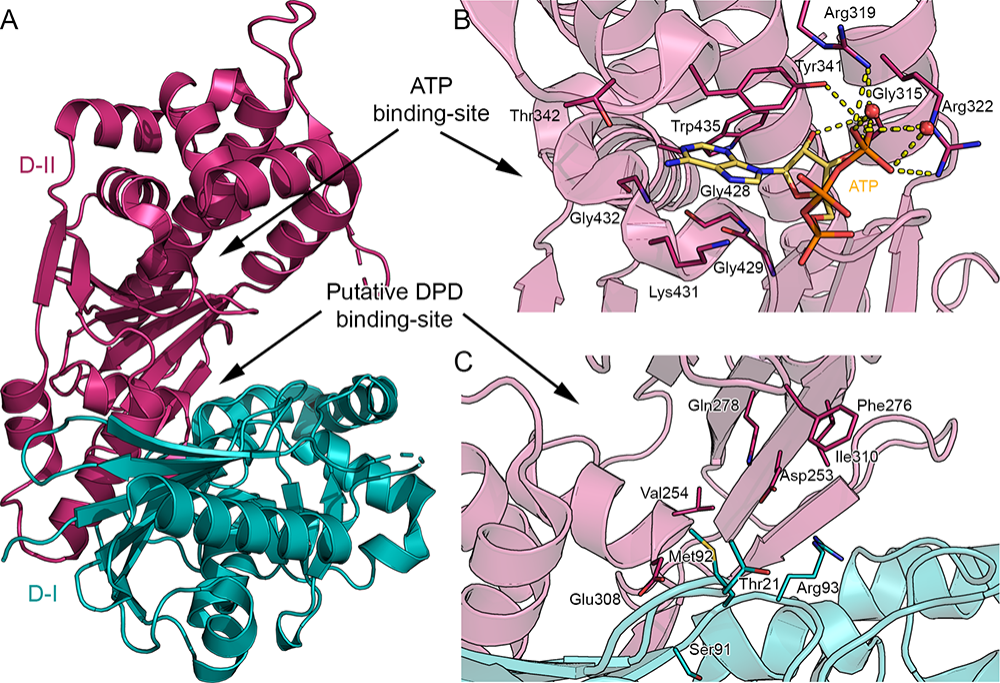
(A) Crystal structure of the LsrK (PDB 5YA1). The two domains D-I and D-II of Lsrk, are visualized as cartoons in teal and magenta, respectively. (B) Close-up of the ATP binding site. ATP is represented in stick mode, with carbon atoms in yellow. (C) Close-up of the putative DPD binding site. Amino acid residues are visualized in stick mode with D-I’s and D-II’s carbon atoms in teal and magenta, respectively. Yellow dashed lines represent the hydrogen bonds. Heteroatoms are color-coded (oxygen atoms in red, nitrogen atoms in blue, sulfur atoms in yellow, phosphorus atoms in orange).

Focusing on LsrK, the kinase’s overall structure can be divided into two domains: the N-terminal domain (or domain I, D-I, Figure 5A) and the C-terminal domain (or domain II, D-II, Figure 5A). This is in accordance with the FFGY superfamily members’ architecture and the results of the homology model studies developed by Medarametla et al.^93^ The two domains are arranged like the valves of a clamp; the joint is articulated between the α-helices 17 of D-II and the β-sheet formed by strands 1, 2, 3, and 6 of D-I. Upon substrate binding, a long-range conformational change causes the two valves of the protein to close, thus preventing the entry of the solvent and the priming of the phosphorylation process. Therefore, in analogy to the FGGY carbohydrate kinases, the existence of an open-inactive and a close-active conformational state is also suggested for LsrK.

The three resolved *Ec*LsrK crystallographic structures portrayed the kinase in its open-inactive conformation. All the cocrystallization or soaking attempts performed by Ha et al. in the presence of DPD or an AI-2 antagonist were unsuccessful. Therefore, only the apo form of the LsrK-HPr complex and a complex with ATP-ADP were reported. Additional STD-NMR studies performed by Ha et al.^89^ revealed that the LsrK-HPr complex’s affinity was higher for ATP than for DPD, thus supporting the above consideration. These experimental observations agree with superimposition studies of the LsrK-HPr complex with the FGGY superfamily member in diverse conformational states (*i.e.*, *ec*XK or *ec*GK), confirming the open-inactive conformation for the newly resolved LsrK-HPr complexes.

The active site of LsrK is located at the cleft between the two domains and contains both the site of interaction for the ATP and the substrate. The ATP site (Figure 5B) is easily identifiable because, in two of the three deposited LsrK X-ray structures, the enzyme is in a ternary complex with ATP (PDB 5YA1) or ADP (PDB 5YA2). ATP and ADP bind near the cleft’s opening and interact with residues in domain II, although ATP seems to bind to HisLsrK/HPr more strongly than ADP. The ATP binding site is delineated by α-helices 6, 7, 8, 12, and 14. The adenine base of ATP or ADP is located deep within a hydrophobic pocket formed by Gly428, Gly429, Lys431, Gly432, Trp435, Thr342, and Tyr341. The hydroxyl in position 4 of the ribose forms an H-bond with Gly315. Lastly, the phosphate group of ATP/ADP is exposed to the solvent, and it is involved in a dense network of H-bonds and salt-bridges with Arg319, Arg322, and the solvent (Figure 5B).

The LsrK/HPr crystal structure has been obtained without including DPD or an AI-2 antagonist. To date, the putative binding site of the substrate was only predicted by homology models developed by Medarametla et al.,^93^ based on the crystallographic structures of *Ec*GK (PDB 1GLF) and *Ec*l-rhamnulose kinase (PDB 2CGJ) in complex with ADP and glycerol or fructose, respectively (employed as a template for the closed conformation), and on the crystallographic structure of *Ec*XK in complex with xylulose (PDB 3HZ6; used as a template for the open conformation). The substrate’s putative binding site is located deeply within the cleft formed by D-I and D-II (Figure 5C). It is enclosed by residues of the two subunits such as Thr21, Ser91, Met92, Arg93, Asp253, Val254, Phe276, Gln278, Glu308, and Ile310 (Figure 5C). Structure-based sequence alignment shows that both the residues of the ATP-binding site as well as the residues of the putative DPD binding site are well conserved among six selected Gram-negative and Gram-positive bacteria (*E. coli*, *Salmonella typhimurium*, *Yersinia pestis*, *Klebsiella pneumoniae*, *Bacillus subtilis*, *Bacillus thuringiensis*, and *Streptococcus* sp.). Because the LsrK binding site pocket depends on the kinase’s conformation, the design of effective and specific inhibitors remains challenging. In-depth knowledge of the catalytic mechanism and its variability concerning substrate binding is necessary to successfully guide the rational design of clinically useful inhibitors.

## In Vitro Assays for the Evaluation of LsrK Activity

**Figure 6:**
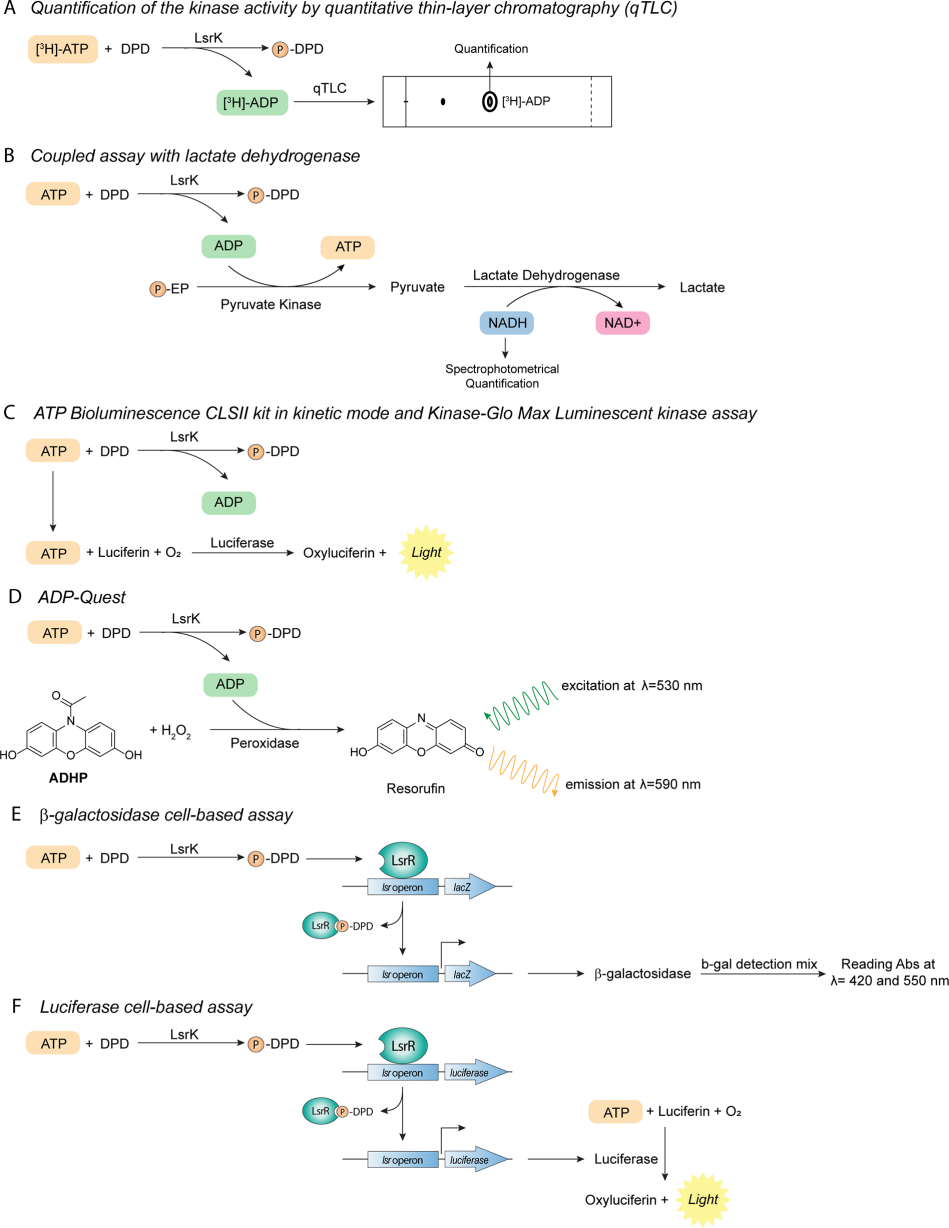
Target-based and cell-based in vitro biochemical assays developed so far for quantifying the LsrK inhibition and its effect on QS.

During the last two decades, several methods and assays have been developed to evaluate the biological activity of LsrK and the testing of small molecules for their inhibitory potential on the kinase (Table 1). In the beginning, the considered kinase activity was assessed by quantitative thin-layer chromatography (qTLC). This is a well-consolidated *in vitro* phosphorylation technique.^94,95^ Briefly, to monitor the conversion of ATP to ADP, the kinase is incubated with potential substrates and radiolabeled ATP, and the resulting labeled ADP is quantified by qTLC (Figure 6A). This method was also exploited to evaluate the ability of LsrK to phosphorylate AI-2,^94^ and to clarify its mechanism of action.^88,90^ Despite the promising results obtained with the qTLC phosphorylation assay, this technique has severe drawbacks. It is expensive, and it requires extra precautions and equipment due to radiolabeled chemicals, and it is not suitable for high throughput screening.

**Table 1:** PROs and CONs of the Biochemical Assay for Monitoring and Quantifying LsrK Activity Developed so Far

biochemical assay	type of screening	PROs	CONs
qTLC	target-based	- direct assay	- poor precision
		- reproducibility	- expensive
		- simple and time-saving	- use of radiolabeled chemicals
		- minimum types of equipment used	- not suitable for HTS

lactate dehydrogenase	target-based	- spectrophotometric assay	- coupled assay
		- kinetic study of LsrK activity	- purified recombinant LsrK protein needed
		- economic	- interference with phosphatases
		- no use of radiolabeled chemicals	

ATP Bioluminescence CLSII kit and Kinase-Glo Max Luminescent kinase kit	target-based	- bioluminescence assay	- expensive
		- extremely sensitive	- high purity of recombinant LsrK protein needed
		- fast and easy to carry out	- sensitive to all the ATP present in the cell-culture
		- suitable for HTS	- “signal decreaseassay”
		- easy to run with high ATP concentrations as a way of selecting against ATP-competitive inhibitors	- sensitive to luciferase inhibitors

ADP-Quest	target-based	- end point or kinetic mode	- coupled assay
		- convenient gain-of-signal with the respect of the ATP-detecting assay	- high purity of recombinant LsrK protein needed
		- detection around 590 nM, which tends to be less susceptible to inner filter effects	

β-galactosidase-based assay	cell-based	- rapid, sensitive, and consistent quantitation of β-galactosidase using a single-reagent addition	- time-consuming
		- performed in either 96- or 384-well plates	- cumbersome when processing large numbers of samples
		- performed in both lysogeny broth (LB) and phosphate-buffered saline (PBS) media	- unspecific β-galactosidase inhibitory activity
			- not functional in glucose-containing media, due to repression of *lsr* operon by glucose
			- the results obtained in one bacterial species cannot always be extrapolated to the other species with the QS system of the same type

luciferase-based assay	cell-based	- fast and simple	- assay kit not commercially available
		- highly sensitive	
		- suitable for HTS	

A reliable alternative for the study of the kinetic activity of LsrK is a spectrophotometric assay reported by Zhu et al. Adenosine diphosphate (ADP), produced as a result of the LsrK catalysis, activates the pyruvate kinase to produce pyruvate that is further metabolized by lactate dehydrogenase with consumption of NADH which could be measured spectrophotometrically to determinate the initial velocity of LsrK catalysis (Figure 6B).^84^

During the last years, several assay kits have been developed based on either luminescence or fluorescence. The ATP bioluminescence CLSII kit in kinetic mode (Roche Scientific, Germany) is a bioluminescence-based method that exploits luciferase activity. Luciferase requires ATP as a substrate to produce light (Figure 6C). In the presence of LsrK, the amount of available ATP (and hence the amount of light generated by luciferase) depends on the activity of LsrK, thus enabling the measurement of LsrK inhibition by small molecules.^96^ Another luminescence-based method is the Kinase-Glo Max Luminescent kinase assay (Sigma-Aldrich, USA). It correlates ATP concentration after phosphorylation with the amount of emitted light (Figure 6C). This assay’s advantages include a more stable luminescence signal and shorter analysis time, making it suitable for the screen of large compound libraries. It also allows higher ATP concentrations (up to 500 μM), thus making it more versatile.^71,93,97,98^ ADP-Quest differs from the two kits described above as it measures fluorescence. ADP-Quest exploits the ADP produced in the kinase reaction to convert 10-acetyl-3,7-dihydroxyphenoxazine (ADHP), a fluorescent dye precursor the fluorescent resorufin. ADHP is then transformed into the fluorescent molecule (λ = 530 nm for excitation, and λ = 590 nm for emission). Peroxidase catalyzes the latter reaction in the presence of hydrogen peroxide. The resulting fluorescence is directly proportional to the activity of the enzyme (Figure 6D). ADP-Quest is suitable for high-throughput screening.^88,99^

The assays described above are often coupled with other methods. For example, thermal shift assays are usually performed to confirm the direct interaction of modulators with LsrK.^93,97^ Same answers can also be obtained from microscale thermophoresis assays.^97^ Furthermore, to evaluate potential modulators’ specificity, compounds can be tested with glycerokinase, an enzyme sharing a similarity with LsrK. The same kits and the same conditions used to evaluate LsrK modulation can be used to assess this property.

To study LsrK modulation’s effect, QS inhibition can also be evaluated on the whole cell in cellular-based models. This assay is based on the measurement of the level of β-galactosidase activity controlled by the *lsr* promoter. LsrK phosphorylates DPD, and the produced P-DPD activates the *lsr* transcription with the production of β-galactosidase that can be quantified through a specific spectrofluorometric assay (Figure 6E).^100^

Gatta et al. have recently developed a new cell-based assay by engineering *E. coli* strain with a bacterial luciferase operon *luxABCDE* under the control of *lsr*. Thus, when AI-2 activates the QS, the *lux* expression is induced with the final production of light, whereas in the presence of an LsrK inhibitor and/or a QS quenching agent, the expression of the luciferase is inhibited with no production of light (Figure 6F).^101^

## LsrK Inhibitors

**Figure 7:**
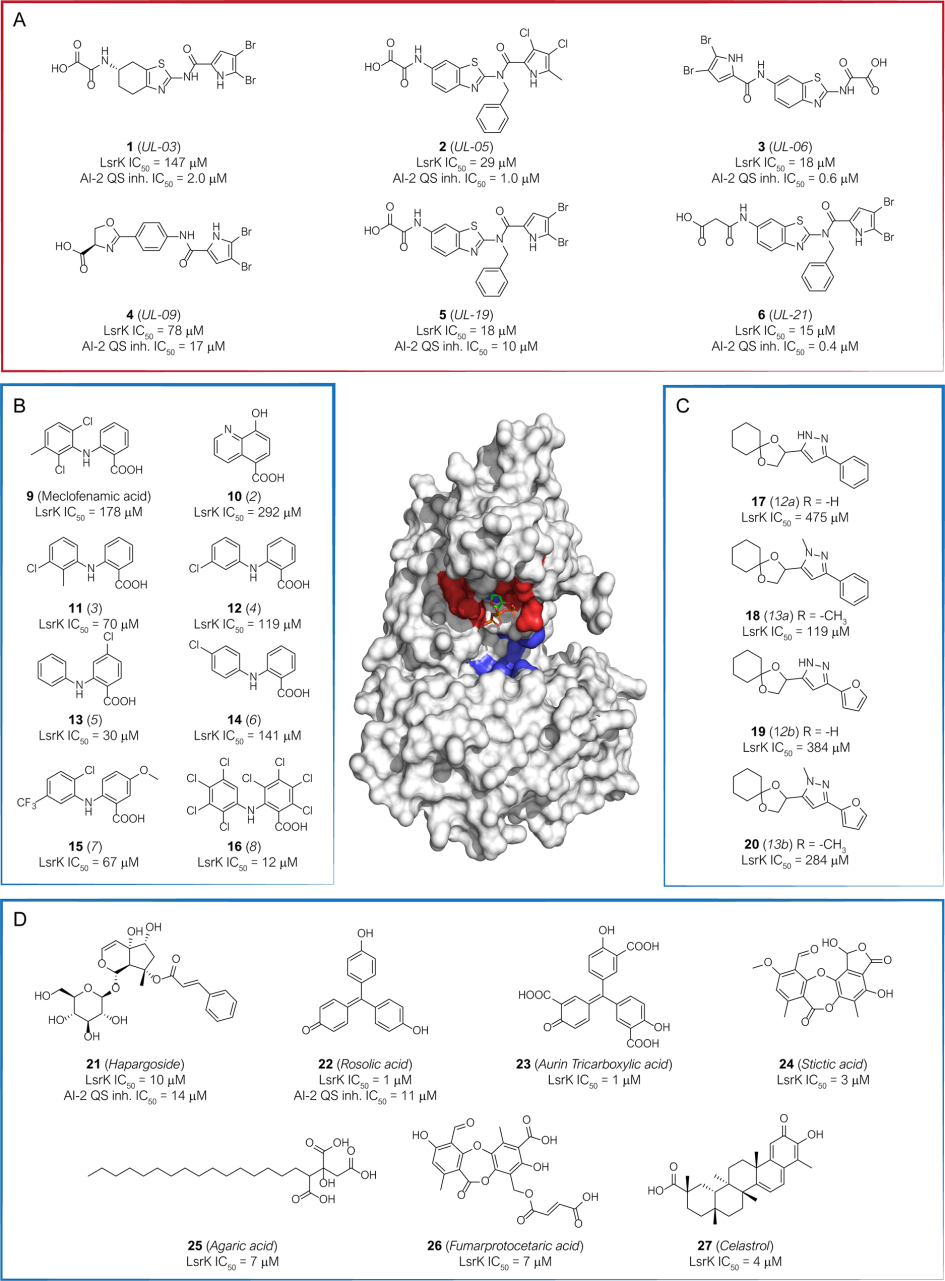
Chemical structure, LsrK inhibitory activity, and AI-2 QS inhibitory activity of the hit compounds identified as LsrK inhibitors so far and mainly discussed in this Perspective. The original numbering of the compounds in the parent paper or the common name for natural compounds is reported in the brackets. The inhibitors directed toward the ATP-binding site (surface colored red in the 3D structure of the protein) are enclosed in the red box. The inhibitors directed toward the putative DPD binding site (surface blue colored in the protein’s 3D structure) are enclosed in blue boxes. (A) Primary hits targeting the ATP-binding site, identified by combining target-based and the new luminescent cell-based assay. (B) Hits identified by structure-based virtual screening. (C) DPD-inspired heterocyclic compounds designed in a structure-based approach. (D) Natural and synthetic hits identified by target-based HTS.

As stated above, LsrK is almost unexplored from a medicinal chemistry standpoint, and only a few compounds have been identified as LsrK inhibitors (Figure 7).

Gatta et al. reported the identification of a set of LsrK inhibitors with the capability to target the ATP binding pocket of the kinase (compounds **1**–**6**, Figure 7A).^101^ The compounds were fished out from a library of 91 compounds originally designed as ATP-competitive gyrase B inhibitors. The library was first screened against LsrK in a target-based assay and 29 primary hits with IC_50_ values ranging from 8 to 147 μM. The entire library was further assessed for AI-2-mediated QS interference using the new cell-based assay based on luminescence and developed by the same research group. The results obtained from the two assays were compared. Six primary LsrK inhibitors (**1**–**6**, Figure 7A) were able to inhibit the QS activation in the cell-based assay with low-sub micromolar IC_50_ (ranging from 0.4 to 17 μM). Interestingly, this cell-based assay fished out additional 18 hits with the potential capability to target other components of the *lsr* pathway.^101^

**Figure 8:**
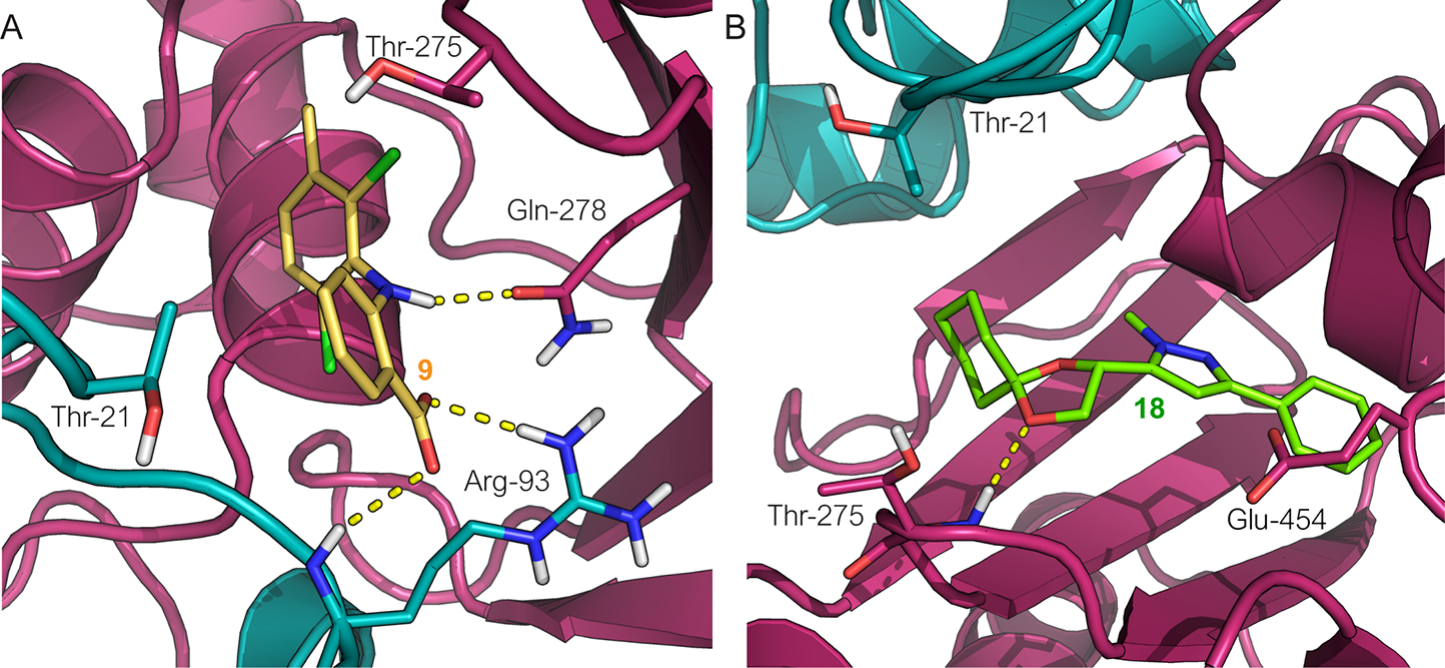
Predicted docking pose of (A) compound **9** (in yellow stick carbon) and of (B) compound **18** (in green stick carbon) at the putative binding site of DPD. Amino acid residues are visualized in stick mode with D-I’s and D-II’s carbon atoms in teal and magenta, respectively. Yellow dashed lines represent the hydrogen bonds. Heteroatoms are color-coded (oxygen atoms in red, nitrogen atoms in blue, sulfur atoms in yellow, phosphorus atoms in orange).

Medarametla et al. were the first to report a structure-based virtual screening study to identify new AI-2s structurally unrelated LsrK inhibitors and directed to the DPD binding site.^93^ In the absence of detailed information on the three-dimensional structure of LsrK, a homology model for predicting the kinase’s 3D structure was developed first. Four models of LsrK were developed, taking into account the open and closed conformation and the inclusion or exclusion of ATP. Virtual screening performed on a library of 132 566 compounds resulted in 104 compounds to be in vitro assessed. The primary screening led to identifying two hits compounds (**9**, namely meclofenamic acid and **10**) with an LrsK IC_50_ of 178 and 292 μM, respectively. The two compounds were further used as templates for searching other analogues by catalogue approach. Fourteen commercially available derivatives were selected and assessed for LsrK inhibitor activity, resulting in six derivatives of compound **9** (compounds **11**–**16**, Figure 7B) with IC_50_ ranging from 12 to 141 μM. A thermal shift assay confirmed the binding of the identified hit to LsrK. Docking of the selected hits suggested an interaction of the compounds with the putative DPD binding site and highlighted potential interactions with the catalytic residues (Arg93, Gln278, and Thr21) that could be exploited for the design of improved inhibitors (Figure 8A).

**Figure 9:**
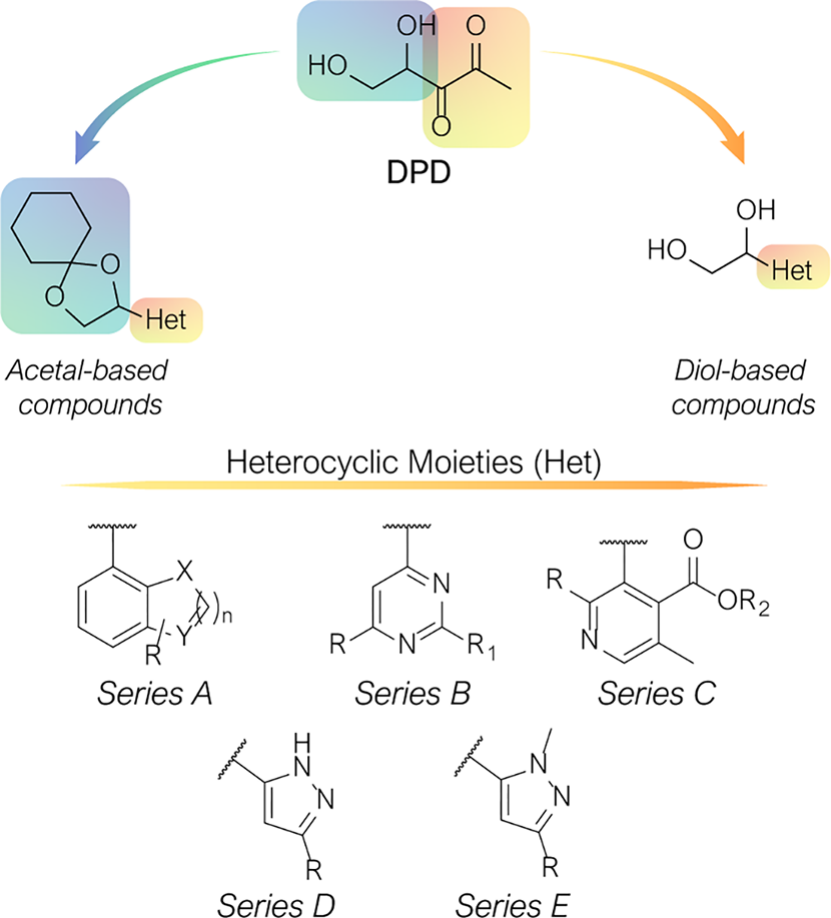
DPD-inspired heterocyclic compounds.

To identify LsrK inhibitors with a chemical structure distinctive from native DPD, Stotani et al., in a structure-based approach, designed, synthesized, and assessed in vitro the inhibition of LsrK by five small libraries of DPD-inspired heterocyclic derivatives (Het-DPD, Figure 9).^70,71^ Taking together the information achieved from a structure–activity relationship (SAR) studies around the main backbone of DPD, a spyrocyclohexyl-dioxolane moiety replaced the portion essential for LsrK-mediated phosphorylation (*i.e.*, the two hydroxyl groups at C4 and C5). In contrast, the diketo group of DPD was embedded in heteroaromatic rings (such as pyrimidine, pyrazole, pyridine, and annulated pyrimidine, Figure 7C).

Among the entire library of compounds investigated by Stotani et al., the pyrazole-containing DPD derivatives **17**, **18**, **19**, and **20** (Figure 7C) emerged as the most promising LsrK inhibitors, with low/medium micromolar IC_50_ against the purified enzyme (475, 384, 119, and 284 μM, respectively). Docking of these compounds to the putative DPD-binding site was performed using the newly released structure of LsrK bound with ATP (PDB 5YA1).^71^ From docking calculation, the DPD diol’s protection in a spyro cyclohexyl-dioxolane moiety is essential for activity because, to accommodate the aryl-pyrazole core moiety, the cyclohexyl ring is positioned into the hydrophobic pocket delimited by Thr21 and Phe267, whereas the dioxolane ring establishes electrostatic interaction with the near Thr275 polar residues (Figure 8B). Conversely, the analogues carrying the free diol moiety resulted in less active because they are forced to orient the two hydroxyl groups toward the negative electrostatic potential surface of the binding site formed by Glu454 and Thr456, resulting in an unfavorable repulsion effect.

Interestingly, among the heterocyclic scaffolds explored, solely the pyrazole derivatives resulted in an effective inhibitor of LsrK probably due to the favorable H-bond interactions that the pyrazole ring can establish with critical residues of the binding site. Thus, the simultaneous presence of the dioxolane ring, the pyrazole moiety, and the protection of the diols as acetal seems necessary for LsrK inhibitor activity. The updated SAR considerations might be exploited in the design of other optimized Het-DPD derivatives.

Gatta et al. developed a reliable and robust target-based HTS-assay for the identification of new LsrK inhibitors.^97^ This methodology was applied to a library of 2000 chemical compounds containing 50% of known drugs, 30% of natural compounds, and 20% of other bioactive compounds. At the end of the screening campaign, 12 compounds with an LsrK IC_50_ < 10 μM were identified. According to the toxicological and physicochemical studies reported in the literature, eight compounds were discarded for promiscuous activity, toxicity, and impaired chemical-physical properties. Harpagoside (**21**, LsrK IC_50_ = 10 μM), rosolic acid (**22**, LsrK IC_50_ = 1 μM), aurin tricarboxylic acid (**23**, LsrK IC_50_ = 1 μM), and agaric acid (**25**, LsrK IC_50_ = 7 μM) were further assessed by thermal shift, confirming the binding to LsrK for the first three compounds (Figure 7D). The capability of the three identified compounds to interfere with the QS cascade was assessed in *E. coli* cell-cultures through the β-galactosidase assay (Figure 6E). **21** and **22** showed an IC_50_ of 11 and 14 μM, respectively. Interestingly, **21** is a metabolite produced by *Harpagophytum procumbens*, popularly known as devil’s claw, used in African traditional medicine as an anti-inflammatory and antirheumatic drug. Moreover, besides **21** and **25**, other natural compounds such as stictic acid (**24**), fumarprotocetaric acid (**26**), and celastrol (**27**, from *Tripeterygium wilfordii*) resulted from the HTS as promising LsrK inhibitors with IC_50_ of 3, 7, and 3 μM, respectively (Figure 7). Of note, the last two compounds are both produced by many lichen species, which makes these organisms fascinating sources of LsrK inhibitors.^102−105^

## Current Challenges and Future Perspectives

The WHO has identified AMR as a significant threat to public health and the economy. Superbug bacteria are responsible for about 25% of infections and almost 30% of AMR-related deaths. Conventional antibacterial drugs usually interfere with bacterial cell wall biosynthesis, protein synthesis, or DNA replication, and the search for new antimicrobials is still mainly focused on these approaches. However, the massive evolutive pressure, consequent to the misuse of antibiotics performed in the last decades, forced bacteria to develop strategies to overcome the antibiotics’ efficacy. Thus, the hunt for new targets and innovative weapons to treat these superbug infections has become a priority. Although the WHO delineated global strategies to contain the spread of resistance, funding, and lack of resources remain the significant obstacles in achieving this goal.^106^ For cell–cell communication, bacteria use QS signaling, a mechanism that allows them to release and detect extracellular signals. As the QS signaling system is involved in bacterial resistance development, QS inhibition could become a new promising antibacterial strategy to prevent bacterial resistance and repress the expression of virulence factor genes related to population density. These virulence factors are not essential for bacterial growth and survival. Thus, focused treatment would not inhibit bacterial growth, not generate selective pressure, and, therefore, is expected to be associated with a much lower risk of resistance development. Advances in the research of QS may lead to the development of novel antibacterial compounds with new modes of action and, in consequence, may provide a paradigm shift in the fight against AMR. In the entire QS process, the bacterial LsrK kinase plays a key role, representing an attractive target. The relationship between LsrK and AMR has been well established. Thus, the exploitation of LsrK as a target to develop drugs to be used alone or in combination with current antimicrobials is at a pioneering stage and may represent an innovative strategy for fighting AMR.

Since the LsrK pathway has been discovered relatively recently, LsrK is still poorly explored from a pharmaceutical standpoint. At present, only a few molecules showing significant interactions with LsrK are known. In this scenario, nature-aided drug discovery can be considered a successful approach to discovering new LsrK inhibitors. Thus, results reported by Gatta et al. in 2019 pave the way to identify new hits from nature. Indeed, as a future perspective, the over 600 iridoid glycosides belonging class of harpagoside and identified in 57 families of different plants can be assessed for inhibition of LsrK.^107^ On the other hand, both fumarprotocetraric acid and stictic acid belong to the class of depsidones, which are ubiquitous metabolites in lichens world. These data should push researchers to consider superior plants and lichens as exciting and innovative sources of active metabolites. However, this research field is still in its infancy.

Although the knowledge about LsrK structure is still limited, the pilot studies described in this perspective offer an overview of the protein’s behavior and highlight all the elements essential to understand better the protein–substrate dynamics and how to interfere with it. The comprehensive consideration of LsrK and related pathways may be an important strategy in developing a new generation of antimicrobials with unprecedented modes of actions and complementary to the currently available antibacterial agents. This strategy could allow fighting the projected worldwide occurrences of superbugs infections. This approach will be novel and nonincremental. The ability of this new generation of antimicrobial drug candidates to surpass any current or in-development technological paradigm is summarized in the following highlights:

- The identified lead compounds will divert from the conventional bacterial targets exploited by pharmaceutical companies to develop antibacterial drugs approved for humans.
- They will not directly kill the bacteria by inhibiting vital targets, but they will attenuate their virulence by interfering with their ability to communicate and organize in communities.
- They will not promote the AMR phenomenon as they will not act on vital targets for bacteria but instead interfere directly with the origin’s resistance development process.
- They may restore the efficacy of currently available antibacterial drugs against resistant bacterial strains through their coadministration.

An in-depth structural insight into the kinase’s structure and function, the generation of a portfolio of LsrK inhibitors, and the consequent in vivo demonstration of the engagement between LsrK and AMR will represent a breakthrough in the fight against antimicrobial resistance.

## Abbreviations Used

2-AA: 2-amino acetophenone
6PGD: 6-phosphogluconate dehydrogenase
6PLG: 6-phosphogluconolactonase
ADHP: 10-acetyl-3,7-dihydroxyphenoxazine
ADP: adenosine diphosphate
AHL: *N*-acyl homoserine lactones
AI-2: autoinducer-2
AMR: antimicrobial resistance
ATP: adenosine triphosphate
cAMP: cyclic adenosine monophosphate
CCR: carbon catabolite repression
CRP: cAMP receptor protein
DHAP: dihydroxyacetone phosphate
DSF: diffusible signal factor
EI: enzyme E I
EII: enzyme E II
EP: enolpyruvate
G6PD: glucose-6-phosphate dehydrogenase
GLASS: Global Antimicrobial Resistance Surveillance System
HPr: histidine protein
LuxS: *S*-ribosylhomocysteinase
P-DPD: *S*-3,3,4,5-tetrahydroxy-2-pentanone-5-phosphate
PEP: phosphoenol-pyruvate
Pfs: 5′-methylthioadenosine nucleosidase
PQS: *Pseudomonas* quinolone signal
P-TPO: 3,4,4-trihydroxy-2-pentanone-5-phosphate
PTS: dependent sugar phosphotransferase system
QS: quorum sensing
qTLC: thin-layer chromatography
*R*-DHMF: (2*R*,4*S*)-2,4-dihydroxy-2-methyldihydrofuran-3-one
*R*-THMF: (2*R*,4*S*)-2-methyl-2,3,3,4- tetrahydroxytetrahydrofuran
Ru5P: ribulose 5-phosphate
SAH: *S*-adenosylhomocysteine
SAM: *S*-adenosylmethionine
*S*-DHMF: (2*S*,4*S*)-2,4-dihydroxy-2-methyldihydrofuran-3one
*S*-DPD: *S*-4,5-dihydroxy-2,3-pentanedione
SRH: *S*-ribosylhomocisteine
*S*-THMF: (2*S*,4*S*)-2-methyl-2,3,3,4-tetrahydroxytetrahydrofuran
*S*-THMF-borate: (2*S*,4*S*)-2-methyl-2,3,3,4-tetrahydroxytetrahydrofuranborate
*S*-THP: *S*-3,3,4,5-tetrahydroxy-2-pentanone
WHO: World Health Organization
